# A Case of Mild Encephalopathy with a Reversible Splenial Lesion Associated with G5P[6]Rotavirus Infection

**DOI:** 10.1155/2013/197163

**Published:** 2013-11-13

**Authors:** Tsuyoshi Matsuoka, Toshifumi Yodoshi, Misaki Sugai, Masato Hiyane, Takashi Matsuoka, Hideki Akeda, Masaharu Ohfu, Satoshi Komoto, Koki Taniguchi

**Affiliations:** ^1^Division of Pediatric Neurology & General, Okinawa Prefectural Nanbu Medical Center & Children's Medical Center, Japan; ^2^Department of Virology and Parasitology, Fujita Health University School of Medicine, Japan

## Abstract

We report a case of mild encephalopathy with a reversible splenial lesion (MERS) associated with acute gastroenteritis caused by rotavirus (RV) infection. The patient (male, 4 years and 3 months old) was admitted to our hospital for diarrhea and afebrile seizures. Head MRI revealed a hyperintense signal in the splenium of the corpus callosum on DWI and a hypointense signal on the ADC-map. After awakening from sedation, the patient's disturbance of consciousness improved. On day 5 after admission of the illness, the patient was discharged from the hospital in a good condition. Electroencephalography on day 2 after admission was normal. On day 8 of admission, head MRI revealed that the splenial lesion had disappeared. RV antigen-positive stools suggested that RV had caused MERS. This RV genotype was considered to be G5P[6]; it may have spread to humans as a strain reassortment through substitution of porcine RV into human RV gene segments. This extremely rare genotype was detected first in Japan and is not covered by existing vaccines; this is the first sample isolated from encephalopathy patients. Few reports have investigated RV genotypes in encephalopathy; we believe that this case is valuable for studying the relationship between genotypes and clinical symptoms.

## 1. Introduction

Tada et al. (2004) reported a case involving clinically mild encephalopathy with a reversible splenial lesion (MERS) [1]. The clinical symptoms of this condition include delirium, disturbance of consciousness, seizures, and vomiting. The splenial lesions show hyperintense signals on diffusion-weighted imaging (DWI) and a low apparent diffusion coefficient (ADC). These lesions characteristically disappear within 1 week. The prognosis of MERS is generally good [2]. We report a case of MERS associated with rotavirus (RV) infection.

## 2. Case Presentation

A male patient aged 4 years and 3 months presented to the ER (emergency room) of our hospital with vomiting, diarrhea, and seizures. On day 1 of the illness, he had developed gastrointestinal symptoms, including vomiting and watery diarrhea that occurred 4-5 times per day. Four days later, bilateral generalized tonic-clonic seizures began, for which he was transported to our hospital. The seizures lasted up to 1 minute and subsided spontaneously. The patient was in a restless state of consciousness (level E3V4M5 according to the Glasgow Coma Scale and level II-10 according to the Japan Coma Scale). He stared, averted his gaze, continued to cry, and threw objects. This disturbance of consciousness persisted for 7 hours. The patient was sedated with midazolam, and MRI was performed. On awakening after 2 hours, the patient had regained a normal state of consciousness, after which there was no recurrence of disturbance of consciousness or seizures.

**Figure 1:**
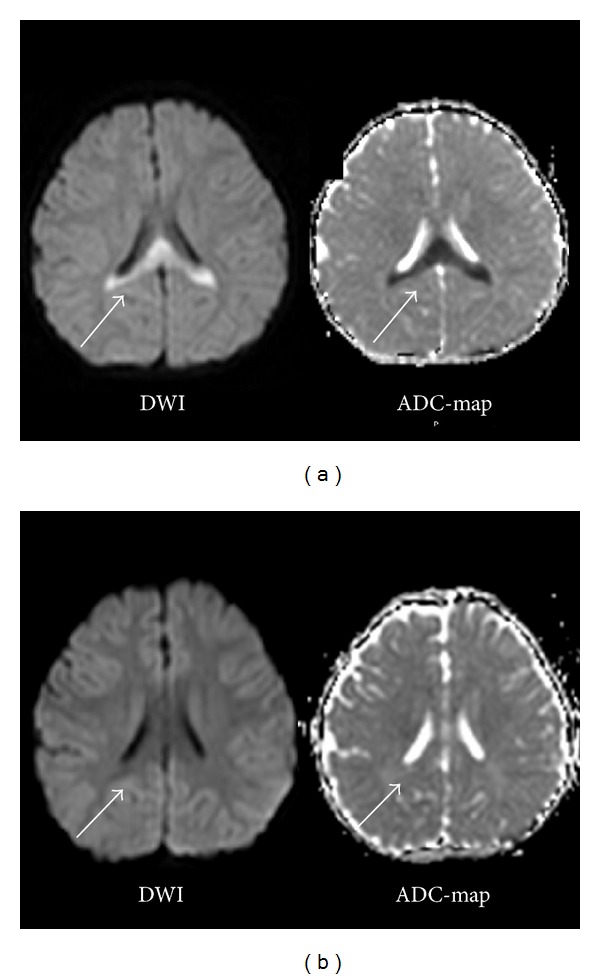
(a) Head MRI on day of admission (day 4 of illness). MRI was performed on the day the seizures and disturbance of consciousness appeared. DWI reveals a hyperintense signal in the splenium of the corpus callosum and a hypointense signal on the ADC-map. (b) Head MRI performed 8 days after admission (day 12 of the illness). Head MRI (DWI, ADC-map) shows that the abnormal signal detected in the splenium of the corpus callosum had disappeared.

On admission, the patient's vital signs were as follows: temperature, 37.7°C; heart rate, 130 beats/minute; blood pressure, 100/60 mmHg; and SpO_2_, 98% (room air). No central nervous system abnormality, meningeal irritation, paralysis, or abnormalities in the thoracoabdominal region were observed. MRI revealed a hyperintense signal in the splenium of the corpus callosum on DWI and a hypointense signal in the ADC-map (Figure 1(a)). The patient's peripheral circulation was good.

The patient's medical history was unremarkable. He was born by spontaneous cephalic delivery after 39 weeks and 3 days of gestation (birth weight, 2664 g). No remarkable observations were noted during the pregnancy or perinatal period. The patient's growth and development were normal. The family medical history revealed that the father had febrile seizures.

The results of the patient's biochemical blood tests were as follows: WBCs, 3900/*μ*L; Hb, 13.4 g/dL; Plt, 26.1 × 10^4^/*μ*L; BUN, 16 mg/dL; Cre, 0.3 mg/dL; Na, 132 mEq/L; K, 4.5 mEq/L; Cl, 101 mEq/L; AST, 41 IU/L; ALT, 21 IU/L; CRP, 2.59 mg/dL; Glu, 84 mg/dL; lactic acid, 7.8 mg/dL; pyruvic acid, 0.32 mg/dL; NH_3_, 49 *μ*g/dL; pH, 7.424; PCO_2_, 39.6 mmHg; HCO_3_, 25.5 mmol/L; and BE, 1.6 mmol/L. No abnormality was detected on blood amino acid analysis or tandem mass screening. Cerebrospinal fluid (CSF) examination revealed a cell count of 4 cells (all mononuclear), a glucose concentration of 95 mg/dL, and a total protein concentration of 16 mg/dL.

On day 2 of admission, an electroencephalogram was obtained during sedated sleep. The spindle wave was appropriate for the patient's age. No bursts were observed. The patient's stool was positive for RV antigen. RT-PCR did not reveal RV RNA in the blood or CSF. RT-PCR analysis of RNA extracted from the stool revealed that the RV genotype was G5P[6].

On day 5 of admission, no recurrences were observed, and the patient was discharged. Eight days after admission, all abnormal signals had disappeared (Figure 1(b)). The clinical course was typical of MERS.

## 3. Discussion

RV is a dsRNA virus belonging to the Reoviridae family. Variability in the genotypes of RV in humans affects the expression of the outer shell proteins VP4 P (proteolytic cleavage protein, “P”) and VP7 G (glycoprotein, “G”) [4]. To date, 27 G genotypes and 35 P genotypes have been confirmed. Of these, 5 GP genotypes account for approximately 90% of human RV infections worldwide: G1P[8], G2P[4], G3P[8], G4P[8], and G9P[8] [6].

In 2006, the administration of 2 types of live vaccines, namely, Rotarix and RotaTeq, was initiated worldwide. These vaccines are effective against the major genotypes of RV [7]. In our patient, the RV genotype isolated from the stool was G5P[6]. Because G5P[6] is extremely rare, the protective effects of existing vaccines have not been investigated.

According to a national survey by Hoshino et al. (2012), 40 (4.0%) of the 983 encephalopathy cases reported between 2007 and 2010 in Japan were caused by RV infection. RV-induced encephalopathy is not uncommon, ranking third after influenza virus (26.6%) and HHV-6 (17.0%). Furthermore, 18 of the encephalopathy cases (45%) caused by RV were MERS. Compared with the incidence of influenza virus (20.2%) and HHV-6 (1.8%), the incidence of MERS is high [3].

Few reports have investigated the genotypes of RV associated with encephalopathy. The genotypes of the 14 RV encephalopathy cases diagnosed between 2005 and 2010 in Japan were all group A RV, with 4 cases of G3, 3 cases of G1, 1 case of G2, and 6 cases untyped (detected from stool samples); all were highly prevalent human RV genotypes. To date, only five G5P[6] RV strains have been isolated worldwide, all of which were detected in human gastroenteritis patients [5, 8]. To the best of our knowledge, this is the first instance of G5P[6] RV detection in an encephalopathy patient.

It is unclear whether RV readily causes encephalopathy, and individual human factors may contribute to the pathogenesis.

This was the first G5P[6] RV sample isolated from an encephalopathy patient.

Moreover, G5P[6] is not covered by existing vaccines. Understanding RV genotypes in encephalopathy is important for defining this pathological condition and enabling the development of a suitable vaccine.

## References

[1] Tada H, Takanashi J, Barkovich AJ (2004). Clinically mild encephalitis/encephalopathy with a reversible splenial lesion. *Neurology*.

[2] Takanashi J, Tada H, Kuroki H, Barkovich AJ (2009). Delirious behavior in influenza is associated with a reversible splenial lesion. *Brain and Development*.

[3] Hoshino A, Saitoh M, Oka A (2012). Epidemiology of acute encephalopathy in Japan, with emphasis on the association of viruses and syndromes. *Brain and Development*.

[4] Estes MK, Kapikian AZ, Knipe DM, Howley PM (2006). Rotaviruses. *Fields Virology*.

[5] Li D-D, Duan Z-J, Zhang Q (2008). Molecular characterization of unusual human G5P[6] rotaviruses identified in China. *Journal of Clinical Virology*.

[6] Santos N, Hoshino Y (2005). Global distribution of rotavirus serotypes/genotypes and its implication for the development and implementation of an effective rotavirus vaccine. *Reviews in Medical Virology*.

[7] Santosham M (2010). Rotavirus vaccine—a powerful tool to combat deaths from diarrhea. *The New England Journal of Medicine*.

[8] Ahmed K, Anh DD, Nakagomi O (2007). Rotavirus G5P[6] in child with diarrhea, Vietnam. *Emerging Infectious Diseases*.

